# Improving care for veterans’ environmental exposure concerns: applications of the consolidated framework for implementation research in program evaluation

**DOI:** 10.1186/s12913-024-10614-y

**Published:** 2024-02-23

**Authors:** Katharine Bloeser, Justin M. Kimber, Susan L. Santos, Chana B. Krupka, Lisa M. McAndrew

**Affiliations:** 1 The War Related Illness and Injury Study Center, The VA New Jersey Health Care System, 385 Tremont Avenue, East Orange, NJ 07018 USA; 2 The Silberman School of Social Work at Hunter College, The City University of New York, New York, NY USA; 3 Buffalo VA Medical Center, Buffalo, NY USA; 4 Russell Sage College, Troy, NY USA; 5 The VA New York Harbor Health Care System, Brooklyn, NY USA

**Keywords:** Environmental exposure, Veterans, Gulf War Illness, CFIR

## Abstract

**Background:**

Healthcare systems, like the US Department of Veterans Affairs (VA), need policies and procedures for delivering care to special populations including those with environmental exposure concerns. Despite being common and pervasive, especially among Veterans, environmental exposures are largely overlooked by healthcare providers. To successfully implement care for Veterans with military environmental exposure concerns, an understanding of contextual factors impeding care on the provider (e.g., knowledge and beliefs) and organizational (e.g., leadership’s priorities) level is needed. Our goal was to conduct an operational needs assessment of providers to examine provider educational needs regarding Veterans’ military environmental exposure concerns.

**Methods:**

In 2020, we surveyed 2,775 VA medical and behavioral health providers. Our cross-sectional assessment was informed by the Consolidated Framework for Implementation Research (CFIR) and assessed barriers and facilitators to the uptake and application of knowledge regarding interdisciplinary care for environmental exposure concerns. The web-based survey was emailed to providers across the United States representing a variety of disciplines and practice settings to reflect the interdisciplinary approach to care for environmental exposures. We used bivariate statistics to investigate the intervention setting, inner setting, and individual characteristics of providers regarding care for environmental exposure concerns.

**Results:**

Approximately one-third of VA medical and behavioral health clinicians report low to no knowledge of environmental exposure concerns. We find 88% of medical and 91% of behavioral health providers report they are ready to learn more about environmental exposures. Half of medical and behavioral health providers report they have access to information on environmental exposures and less than half report care for environmental exposures is a priority where they practice.

**Conclusions:**

Our findings suggest interdisciplinary providers’ knowledge of and discussion with Veterans about environmental exposures may be influenced by contextual factors at the organizational level. Considering individual-level factors and organizational culture is important to consider when supporting care for environmental exposures. Since this needs assessment, VA established targeted programs to improve care related to military environmental exposures in response to legislation; future exploration of these same variables or contextual factors is warranted.

## Background

Every healthcare system must address the specialty needs of populations they serve (e.g., sexual and gender minorities, and older adults), but are often unsuccessful. The healthcare needs of distinct populations go overlooked for many reasons—lack of provider knowledge [1], leadership does not feel concerns warrant special attention [2], or failure of policy makers to note their significance [3]. Addressing the effects of military environmental exposures, such as toxic substances (e.g., Agent Orange [4]) and airborne hazards (e.g., sand, dust, particulate matter [5]), are central to the healthcare needs of Veterans. Despite efforts to educate and train medical, behavioral health, and dedicated on-site specialists in the form of environmental health clinicians, research and evaluation efforts demonstrate that Veterans often do not receive adequate interdisciplinary care for environmental exposure concerns [6–9]. Activism from Veteran groups [10] and directives from the government [11], including recent legislation, have resulted in an opportunity for the US Department of Veterans Affairs (VA), to improve care for environmental exposure concerns. To successfully implement interdisciplinary care for Veterans with environmental exposure concerns, a baseline understanding of facility and provider level contextual factors contributing to needs on the provider (e.g., knowledge and beliefs) and organizational (e.g., leadership’s priorities) level is warranted [12, 13].

Care for Veterans with concerns related to military environmental exposure is central to the mission of the VA. Vietnam Veterans struggle with health conditions, including cancers, stroke, and type 2 diabetes, related to Agent Orange exposure, an herbicide used throughout the war [4]. In the 1950s through 1980s, primarily Marine Corps Veterans and their families living at Camp Lejeune were exposed to tetrachloroethylene in their drinking water from a nearby dry-cleaning establishment, which is associated with cancers and other chronic diseases [14]. Other garrison (i.e., military post) exposures associated with poor health outcomes include per-and polyfluoroalkyl substances (PFAS), a widely used substance in consumer and commercial products and a component fire-fighting foams used on military bases [15–17]. Gulf War Veterans report high rates of health concerns including fatigue, problems with mood, sleep difficulties, and muscle pain related to military environmental exposures during the Gulf War [18]. For Operation Enduring Freedom/Operation Iraqi Freedom/Operation New Dawn (OEF/OIF/OND) era Veterans, one of the marked deployment related health concerns are respiratory symptoms associated with burn-pits (i.e., large pits of burning trash and refuse) or other airborne hazard exposures [5, 19]. Overall, concerns about military environmental exposures are highly prevalent among US Veterans [20].

The VA has worked to increase provider knowledge about military environmental exposures through having at least one environmental health clinician, a subject matter expert, with administrative support from an environmental health coordinator, in each of the VA’s 172 medical centers [21] and through having tertiary specialty care centers. Use of local expertise focused on one clinician (e.g., a facility champion) has been effective in VA to disseminate best practices for other conditions [22]. Environmental health clinicians address Veteran concerns, provide registry exams, or consult with local clinicians [21] while tertiary referral centers like the War Related Illness and Injury Study Center (WRIISC) and Airborne Hazards and Burn Pits Center of Excellence (AHBPCE) offer case consultation and education to providers [23, 24]. These efforts augment the creation and dissemination of clinician education on care for military exposure concerns and clinical practice guidelines aimed at both medical and behavioral health clinicians [14, 23, 25]. VA’s education initiatives focus on improving care for military exposure concerns by increasing clinician awareness of the importance of discussing military exposures with Veterans; and provide knowledge of military exposures and how to care for them. In 2021, VA and the US Department of Defense (DoD) developed a clinical practice guideline (CPG) for Gulf War Illness (GWI), a condition associated with environmental exposures. The CPG directs interdisciplinary care in the form of cognitive behavioral therapy, health behaviors (e.g., diet, exercise), and medications.

While it is likely that these efforts have made improvements to care for exposure concerns, there is evidence that environmental exposures persist as an omitted area of focus in clinical care for behavioral and medical providers [6, 9, 26]. A 2017 needs assessment found the VA needs to better support providers to perform environmental exposure care. At minimum, providers must speak with Veterans about environmental exposures. However, less than half of VA providers across disciplines report speaking with Veterans about airborne hazards (41%) and 57% of VA providers report having no to low knowledge of airborne hazards [9]. To provide interdisciplinary care for environmental exposures, providers must also feel gaining knowledge about exposures is important and then feel confident in their ability to address Veterans’ concerns. An analysis of the contextual factors associated with care for environmental exposures may help bridge the gap between behavioral and medical providers believing care is important and their subsequently providing interdisciplinary care for military exposures to Veterans.

The Consolidated Framework for Implementation Research (CFIR) [26] can guide the implementation of future interventions and evaluate the current climate for care related to environmental exposures. The CFIR framework often focuses on specific interventions (e.g., screening tools or guidelines). Care for environmental exposures is more nascent; evaluations suggests that providers do not broach the subject of environmental exposures with Veterans, and thus are likely not using current clinical practice guidelines [9, 25]. The current evaluation project focuses on identifying the contextual factors that influence care for environmental exposures in VA. Providing adequate care for environmental exposure concerns includes providers’ perceiving care for environmental exposures as important for improving Veteran health, providers asking about environmental exposures, and providers learning about possible care for related conditions through training [9]. The CFIR uses five domains to operationalize contextual factors: analysis of the intervention characteristics (e.g., perceived efficacy of the care for exposure concerns), the outer setting (e.g., outside groups or policies support care for exposure concerns), the inner setting (e.g., the organization sees care for exposure concerns as a priority), characteristics of individuals (e.g., providers think that care for exposure concerns is important), and the implementation process (e.g., the appropriate people engaged in the development and evaluation of the intervention) [13, 26].

The CFIR model emphasizes how the intervention, or care approach, was developed and by whom as well as the context of its implementation. [27] The CFIR has been used to center evaluation on provider (i.e., individual characteristics) and organization-level (i.e., inner setting) factors that influence successful implementation [28]. The current inquiry seeks to further understand these contexts, specifically how frontline providers perceive intervention characteristics (i.e., the value of care for environmental exposures), the inner setting, and individual characteristics and how these domains influence key outcomes. It also tries to understand how these factors differ between medical providers, such as physicians, behavioral health providers, such as social workers and psychologists, and environmental health clinicians, the VA’s designated specialists for environmental exposures. We pose the following: (1) Do clinical provider perceptions of care for exposure concerns, frequency of discussions with Veterans about exposure concerns, beliefs about education for exposure concerns, and current knowledge of environmental exposures differ by provider type? (2) How do clinical providers assess CFIR domains (i.e., the intervention, inner setting, and individual characteristics) regarding care for environmental exposure concerns at their VA facility? Do these differ by clinical provider type? Findings hold implications for understanding contextual factors to target to improve care for military exposures in the VA.

## Methods

This endeavor was part of a larger national needs assessment initiated by a VA specialty care center and the VA Institute for Learning, Education and Development (ILEAD) to assess the learning needs and priorities of VA providers. In 2020, the needs assessment link was emailed to between 12,000 and 13,000 VA employees across the country representing a variety of disciplines and practice settings to reflect the interdisciplinary approach to care for environmental exposures. For the current study (*N* = 2,775), we selected responses from medical (*n* = 1,609), behavioral health (*n* = 1,046; 38%), and environmental health (*n* = 120; 4%) providers (see Table 1). The non-clinical or administrative staff (*n* = 957) who responded to the survey were not included in the current analysis. The needs assessment asked respondents about their perceptions of care for military exposures and their perceived education, training, and contextual needs related to military environmental exposure concerns.

### Ethics approval

Consistent with VA Program Guide 1200.21, this project was considered program evaluation, not research, and did not require institutional review board approval. Upon starting the needs assessment, respondents were provided with the reason they were selected to receive the emailed assessment, the purpose of the assessment, the office conducting the assessment, assurance that participation was voluntary and that responses were confidential and contact information for those leading the needs assessment.

### Assessment of non-response bias

A total of 3,732 participants completed the assessment suggesting a response rate of 29-31%; which is consistent with estimated of response rates for unincentivized web-based surveys among professionals [29]. To assess non-response bias, we selected participants who responded to the survey three weeks or later than the initial email invitation (*n* = 1,094) [30]. Note, over approximately one month, email reminders were sent to respondents with the survey link. Analysis of non-response bias suggests that the sample is likely under-representative of primary care physicians (14% of late responders vs. 8% of remainder of sample) and nurse practitioners (10% of late responders vs. 6% of remainder of sample). Data suggested that likely non-respondents (i.e., late responders), as compared to respondents, are not more motivated to care for environmental exposure concerns nor have greater knowledge of airborne hazards.

### Outcome measures

#### Provider discussion of environmental exposure concerns

Providers indicated if they have ever spoken with a Veteran about six environmental exposures/concerns (i.e., GWI, airborne hazards, solvents, garrison exposures, Agent Orange, and exposures at Camp Lejeune) or if this discussion was not relevant to their role. This was recoded into a binary variable (i.e., yes indicated the provider had spoken with a Veteran about the exposure and no indicated the provider has never spoken with a Veteran about the exposure).

#### Provider perceptions of training related to environmental exposures

Providers were asked, “how important completing training in the [following] areas are to your role in providing Veteran health care” on a 5-point Likert scale (1 = Not at all important, 5 = Very important). The areas provided were 6 exposures (GWI, airborne hazards, solvents, garrison exposures, Agent Orange, and exposures at Camp Lejeune).

#### Provider knowledge regarding environmental exposures

Provider knowledge of environmental exposures was assessed using six Likert scale items assessing knowledge of GWI, airborne hazards, solvents, garrison exposures, Agent Orange, and exposures at Camp Lejeune. Providers were asked to rate their knowledge from 1 = No knowledge (none) to 5 = Expert knowledge. Providers also indicated the exposure was not relevant to their role.

### CFIR domain measures

#### Intervention characteristics

Providers indicated their level of agreement with the following statement, “Care for environment health concerns improves Veterans’ health,” using a 5-point Likert scale (1 = Strongly disagree, 5 = Strongly agree).

#### Inner setting

Providers endorsed their level of agreement with statements related to the inner setting including infrastructure, workplace culture, and resources in place to support care for environmental exposures [13]. These assessed local facility provision of care, support for education for, priority for integration of care, and the availability of resources and information related to environmental exposures on a 5-point Likert scale (1 = Strongly disagree, 5 = Strongly agree).

#### Characteristics of individual

Provider-level knowledge and beliefs, attitudes, familiarity with, and motivation to provide care [13] for environmental exposures were assessed using two variables. Providers were asked their level of agreement with the statements, “I am motivated to integrate care for environmental exposures” and “I am ready to learn more about environmental exposures” on a 5-point Likert scale (1 = Strongly disagree, 5 = Strongly agree).

### Analysis

In descriptive analysis, we assessed provider discussion with Veterans regarding, perceptions of training on, and knowledge of environmental exposures by provider type using a series of Chi-square tests for association. We then ran a series of Chi-square analyses to test for association between the three CFIR domains and provider type. All analyses were considered statistically significant at the *p* ≤ 0.05 level and used SPSS version 28.

## Results

### Outcomes: providers and environmental exposures

Of the 2,775 VA providers who responded to the needs assessment, 58% were medical providers, 38% were behavioral providers (e.g., psychologists and social workers), and 4% were (*n* = 120) environmental clinicians. Table 1 shows that medical and behavioral health providers were less likely than specially trained environmental health clinicians to have discussions with Veterans about exposure concerns and less likely to see training in environmental exposures as important to their role. Among medical and behavioral health providers, the most frequently discussed environmental exposure was Agent Orange, with 78% of medical and 72% of behavioral health providers reporting having spoken with a Veteran about the topic. The least frequent discussions were about garrison exposures with 14% of medical providers and 12% of behavioral health providers reporting discussing these exposures with Veterans. Between 50% and 64% of medical and between 43% and 55% of behavioral health providers believed that training on environmental exposures was important to their role in providing health care to Veterans. In comparison, specially trained environmental health clinicians were more likely to agree that training on environmental exposures was important to their role (75-89%).

**Table 1:** Discussion of and perceived importance of training regarding care for environmental exposures among VA providers, *N* = 2,775

	Ever discussed with Veteran				Agree training is important to role			
	Medical Providers *n* = 1,609^a^ n(%)	Behavioral Health Providers *n* = 1,046 n(%)	Env. Health Clinician^b^ *n* = 120 n(%)	χ^2^	Medical Providers *n* = 1,609^a^ n(%)	Behavioral Health Providers *n* = 1,046 n(%)	Env. Health Clinician^b^ *n* = 120 n(%)	χ^2^
Gulf War Illness Airborne Hazards Solvents Garrison Exposures Agent Orange Camp Lejeune	682 (57.9) 596 (51.1) 503 (43.4) 165 (14.2) 909 (78.0) 546 (46.9)	446 (58.1) 358 (47.0) 274 (36.1) 93 (12.2) 545 (71.6) 369 (48.4)	100 (96.2) 100 (95.2) 90 (85.7) 68 (66.0) 101 (97.1) 92 (88.5)	59.87^**^ 86.47^**^ 93.03^**^ 201.85^**^ 36.13^**^ 66.73^**^	684 (55.8) 702 (57.5) 674 (55.3) 616 (50.6) 776 (63.6) 611 (50.1)	420 (53.2) 409 (52.0) 384 (48.8) 348 (44.3) 434 (55.4) 340 (43.5)	95 (88.8) 92 (86.0) 86 (80.4) 79 (74.5) 93 (87.7) 89 (83.2)	49.03^**^ 44.91^**^ 39.37^**^ 35.56^**^ 45.65^**^ 60.03^**^

^a^Primary Care Physicians *n* = 365; Other Physicians *n* = 501; Registered Nurses *n* = 415; Nurse Practitioners and Physicians Assistants *n* = 433

^b^ Primary Care Physicians *n* = 12; Other Physicians *n* = 42; Physicians Assistants *n* = 22; Nurse Practitioners *n* = 29; Other *n* = 15

^**^p < 0.001

Table 2 illustrates provider knowledge of key environmental exposures and their perception of this knowledge as relevant to their role. Providers appeared to have the most knowledge about Agent Orange (46% of medical providers, 40% of behavioral providers, and 80% of environmental health clinicians report average to expert knowledge) and the least knowledge of garrison exposures (12% of medical providers, 8% of behavioral health providers, and 58% of environmental health clinicians report average to expert knowledge).

**Table 2:** Knowledge of environmental exposure concerns among VA providers, *N* = 2,775

	Medical Providers *n* = 1,609^a^ n(%)	Behavioral Health Providers *n* = 1,046 n(%)	Environmental Health Clinician *n* = 120 n(%)	χ^2^ (p-value)
Gulf War Illness				
*No to low knowledge* *Average to expert knowledge* *Not relevant to role*	555 (34.5) 532 (33.1) 522 (32.4)	354 (33.8) 345 (33.0) 347 (33.2)	8 (6.7) 96 (80.0) 16 (13.3)	112.38 (*p* < 0.001)
Airborne Hazards				
*No to low knowledge* *Average to expert knowledge* *Not relevant to role*	498 (31.0) 573 (35.6) 538 (33.4)	396 (37.9) 269 (25.7) 381 (36.4)	11 (9.2) 93 (77.5) 16 (13.3)	137.38 (*p* < 0.001)
Solvents				
*No to low knowledge* *Average to expert knowledge* *Not relevant to role*	653 (40.6) 412 (25.6) 544 (33.8)	468 (44.7) 184 (17.6) 394 (37.7)	21 (17.5) 81 (67.5) 18 (15.0)	148.44 (*p* < 0.001)
Garrison Exposures				
*No to low knowledge* *Average to expert knowledge* *Not relevant to role*	822 (51.1) 195 (12.1) 592 (36.8)	540 (51.6) 87 (8.3) 419 (40.1)	32 (26.7) 70 (58.3) 18 (15.0)	245.48 (*p* < 0.001)
Agent Orange				
*No to low knowledge* *Average to expert knowledge* *Not relevant to role*	368 (22.9) 737 (45.8) 504 (31.3)	290 (27.7) 413 (39.5) 343 (32.8)	5 (4.2) 96 (80.0) 19 (15.8)	76.67 (*p* < 0.001)
Camp Lejeune				
*No to low knowledge* *Average to expert knowledge* *Not relevant to role*	728 (45.2) 332 (20.6) 549 (34.1)	452 (43.2) 233 (22.3) 361 (34.5)	14 (11.7) 87 (72.5) 19 (15.8)	169.90 (*p* < 0.001)

### Contextual factors: intervention characteristics, inner setting, and individual characteristics

Table 3 shows the intervention characteristics (i.e., the belief that care for environmental health concerns improves Veteran health), inner setting (i.e., facility level support for educating providers about, prioritizes integrating care for, and supplying resources and information about environmental exposures), individual characteristics (i.e., motivation to integrate care for and readiness to learn more about environmental exposures).

**Table 3:** Strongly Agree/Agree with CFIR Domain Responses by Provider Type

	Medical Providers *n* = 1,609^a^ n (%)	Behavioral Health Providers *n* = 1,046 n (%)	Environmental Health Clinician *n* = 120^b^ n (%)	χ^2^ (p-value)
**I. Intervention Characteristics**				
Care for environmental health concerns improves Veterans health.	1,316 (94.0)	843 (94.9)	107 (96.4)	2.37 (0.669)
**II. Inner Setting**				
My VA supports educating providers about environmental exposure concerns.	795 (56.8)	535 (60.2)	74 (66.7)	6.88 (0.143)
Integrating care for environmental exposures into healthcare of Veterans is a priority where I practice.	641 (45.8)	418 (47.1)	57 (51.4)	1.98 (0.739)
Resources needed to ensure care for environmental exposures are integrated into the healthcare of Veterans are available to me.	617 (44.1)	418 (47.1)	72 (64.9)	18.57 (*p* < 0.001)
I have access to information about care for environmental exposures.	674 (48.2)	477 (53.8)	92 (82.9)	51.90 (*p* < 0.001)
**III. Individual Characteristics**				
I am motivated to integrate care for environmental exposures into healthcare for Veterans.	1,142 (81.6)	735 (82.9)	100 (90.1)	6.63 (0.157)
I am ready to learn more about environmental exposures.	1,236 (88.3)	811 (91.4)	107 (96.4)	11.50 (0.021)

^a^ PCPs *n* = 365; Other Physicians *n* = 501; RNs *n* = 415; NPs and PAs *n* = 433; ^b^PCPs *n* = 12; Other Physicians *n* = 42; PA *n* = 22; NP *n* = 29; Other *n* = 15

*Intervention Characteristics*: 94-96% of providers believe care for environmental exposures improves Veterans’ health; there was no statistically significant difference by provider type. *Inner Setting*: There was no statistically significant difference between providers’ (including environmental health clinicians) perception that their facility supports educating providers about and prioritizes integrating care for environmental exposures. There was a statistically significant difference between providers’ report of having the resources needed to integrate environmental exposures into their practice available to them (44% of medical and 47% of behavioral health providers) compared to 72% of environmental health clinicians. Similarly, 83% of environmental health clinicians reported having access to information on environmental exposures in comparison to 48% of medical providers and 54% of behavioral health providers. *Individual Characteristics*: Most providers across all types were motivated to integrate care for environmental exposures into health care for Veterans (82-90%) and were ready to learn more about environmental exposures (88-96%).

## Discussion

In a needs assessment conducted in 2020, we found that VA medical and behavioral health providers do not frequently have discussions about environmental exposures and related health conditions with Veterans yet do see training in exposure concerns as important to their role in providing care to Veterans. We also found most VA medical and behavioral health providers either lack knowledge of many environmental exposure concerns or see that knowledge as not relevant to their role. These results support Veterans’ perceptions that medical and behavioral health providers need more knowledge about key military environmental exposures [9, 31]. Our findings are also consistent with previous findings within VA [9, 32] and outside VA suggesting providers do not regularly assess for and know about environmental exposures [33–36]. In terms of specific exposure concerns, VA providers were most likely to have discussed with Veterans or have knowledge of Agent Orange and least likely to have discussed with Veterans or have knowledge of garrison exposures (e.g., PFAS). While Agent Orange is likely the more publicly known exposure among a certain cohort of Veterans [36], garrison exposures impact a larger percentage of the Veteran population. Garrison (i.e., military post or installation) exposures encompass a variety of concerns including exposures to PFAS [17–19], blasts [37], chemical exposures [38], and lead exposure [39].

The VA has worked to improve access to knowledge and expertise for Veterans and their providers, including requiring at least one environmental exposure health clinician with advanced training in military environmental exposures be designated in each medical center [40]. While medical and behavioral health providers are not engaging in key practices related to environmental exposures, we found environmental health clinicians were more likely than other providers to possess and apply knowledge regarding care for environmental exposures. Unfortunately, our findings are consistent with the notion that, without additional support, the efforts of one on-site champion may not impact other types of providers (e.g., primary care clinicians) [20, 41]. For efforts to impact other clinicians, research suggests there needs to be focused support from facility and organizational leadership [42]. Therefore, to effect change for care for military environmental exposures, more needs to be done at the organizational level, such as having an organizational leader (e.g., medical center director) in addition to a clinical champion (e.g., environmental health clinician) [42].

Our application of the CFIR model suggests that barriers to care for environmental exposure concerns persist not at the provider level, but at the facility level. We found that both medical and behavioral health providers see training in environmental exposure concerns as important, are motivated to incorporate care for environmental exposure concerns into their practice, and are ready to learn more. However, providers, including environmental health clinicians, did not agree that their local facility supported education for environmental exposure concerns and did not agree that care for environmental health concerns is a priority where they practice. One model for prioritizing national, regional, and local initiatives and demonstrating the importance of key issues in Veteran health among providers has been universal screening and treatment. This has been successfully demonstrated with military sexual trauma (MST), another prevalent occupational stressor among Veterans. The VA has deployed universal screening for MST paired with efforts to educate frontline providers across disciplines [43]. Local policy and actions (e.g., audit and feedback practices) [44] had a strong impact on the implementation of the national MST universal screening policy. These audit and feedback policies use tools and real-time data on performance can help facilities and providers assess implementation of measures like universal screening [44]. Since this needs assessment was conducted, VA has implemented mandated universal screening and training for military environmental exposures, as required by the Honoring our PACT Act of 2022 (H.R. 3967). The universal Toxic Exposure Screening adds a new tool to track Veterans’ military environmental exposure concerns; VA already uses registries, research studies, or specialty clinic referrals to track environmental exposure concerns among Veterans [5, 45]. These efforts have been coupled with local and national leadership’s dedication to successful and data-driven implementation [46].

The current needs assessment has several limitations. First, this was operations data and thus was not gathered to test hypotheses. Second, we asked providers about their perceptions, but did not have objective measures (e.g., testing knowledge). Furthermore, the measures used mostly consisted of one or two items. There are very few validated measures for the variables studied, especially those related to the CFIR [12]. Third, unlike other applications of the CFIR, [27–29, 47] we did not assess implementation of a concrete evidence-based practice; we focused on preliminary steps to implementing care for environmental exposures. An important next step in this work could involve assessment of evidence-based practices and CPGs as they are developed. Additionally, while the survey was anonymous and through the VA’s ILEAD, which does not oversee clinical care, the survey was administered by a VA entity. As such, providers may have felt uncomfortable expressing their true opinion or lack of knowledge. Future needs assessments can and should track change after passage and implementation of the PACT Act to further inform practice and policy development.

In conclusion, our needs assessment found that medical and behavioral health providers in VA report low knowledge of environmental exposures and related conditions or do not see this knowledge as relevant to their role. While VA providers were motivated to learn more, they did not perceive a facility-level culture that supported care for environmental exposures. Our evaluation suggests efforts to train and deploy environmental health clinicians at medical centers are successful, but their impact may be improved with further institutional and facility-level support. As a result of the PACT Act of 2022, which passed after this needs assessment was completed, VA has rolled out universal screening and required training in military environmental exposures, among other initiatives. [48] The impact of continuing and new initiatives should be examined in future evaluations.

## Data Availability

The datasets generated and analyzed during the current study are not publicly available due to regulatory restrictions but are available from the corresponding author on reasonable request.

## Abbreviations

AHBPCE: Airborne Hazards and Burn Pit Center of Excellence
CPG: Clinical Practice Guideline
CFIR: Comprehensive Framework for Implementation Research
GWI: Gulf War Illness
PACT Act: Honoring our Promise to Address Comprehensive Toxics Act of 2022
ILEAD: Institute for Learning, Education and Development
PFAS: Per-and poly Fluoroalkyl Substances
VA: US Department of Veterans Affairs
WRIISC: War Related Illness and Injury Study Center
